# Exploring the edible gum (galactomannan) biosynthesis and its regulation during pod developmental stages in clusterbean using comparative transcriptomic approach

**DOI:** 10.1038/s41598-021-83507-3

**Published:** 2021-02-17

**Authors:** Sandhya Sharma, Anshika Tyagi, Harsha Srivastava, G. Ramakrishna, Priya Sharma, Amitha Mithra Sevanthi, Amolkumar U. Solanke, Ramavtar Sharma, Nagendra Kumar Singh, Tilak Raj Sharma, Kishor Gaikwad

**Affiliations:** 1 ICAR-National Institute for Plant Biotechnology, New Delhi, India; 2 ICAR-Central Arid Zone Research Institute, Jodhpur, India; 3 DBT-National Agri-Food Biotechnology Institute, Mohali, India

**Keywords:** Biotechnology, Molecular biology, Plant sciences

## Abstract

Galactomannan is a polymer of high economic importance and is extracted from the seed endosperm of clusterbean (*C. tetragonoloba*). In the present study, we worked to reveal the stage-specific galactomannan biosynthesis and its regulation in clusterbean. Combined electron microscopy and biochemical analysis revealed high protein and gum content in RGC-936, while high oil bodies and low gum content in M-83. A comparative transcriptome study was performed between RGC-936 (high gum) and M-83 (low gum) varieties at three developmental stages viz. 25, 39, and 50 days after flowering (DAF). Total 209,525, 375,595 and 255,401 unigenes were found at 25, 39 and 50 DAF respectively. Differentially expressed genes (DEGs) analysis indicated a total of 5147 shared unigenes between the two genotypes. Overall expression levels of transcripts at 39DAF were higher than 50DAF and 25DAF. Besides, 691 (RGC-936) and 188 (M-83) candidate unigenes that encode for enzymes involved in the biosynthesis of galactomannan were identified and analyzed, and 15 key enzyme genes were experimentally validated by quantitative Real-Time PCR. Transcription factor (TF) WRKY was observed to be co-expressed with key genes of galactomannan biosynthesis at 39DAF. We conclude that WRKY might be a potential biotechnological target (subject to functional validation) for developing high gum content varieties.

## Introduction

Clusterbean (Guar) has been accorded a status of an economically important crop because of its high galactomannan content present in the seed endosperm. It is a drought tolerant legume crop having chromosome number 2n = 14. With more than 80% soluble dietary fibre, cluster bean is also a nutritionally significant crop. More than 99% of the guar gum consists of galactomannan, which is a commonly used natural thickener^1^. Besides, it has uses in several industries including pharmaceuticals (for treatment of diverse diseases). India is the largest producer and a major exporter of guar gum^2^.

Structurally, galactomannan consists of serially linked (1 → 4) β-D-mannopyranosyl as backbone with (1 → 6) linked α-D-galactopyranosyl residues as side chains. Three different enzymes are principally involved in galactomannan biosynthesis^3^. Mannan synthase (ManS) produces the mannan polysaccharide backbone^1^, while galactosyltransferase (GMGT) transfers galactosyl residues from UDP-galactose to the mannan backbone for assembly of the galactosyl side chains^4^. Both enzymes work closely and their peak activity occurs around 25–35 days after flowering in the seed endosperm^1,5^. A third enzyme, namely α-galactosidase (α-Gal), regulates the galactose substitution^6^. For galactomannan degradation, the enzymes involved are: (1) α-galactosidase that degrades galactomannan when side-chain of mannan backbone is galactose, (2) endo-β-mannanase, a hemicellulose, which acts on the β-1, 4 linkage between the mannose residues present in the mannan backbone and (3) β-mannosidase (EC 3.2.1.25) an exo-glycosidase which breaks the glycosidic bond of terminal non-reducing mannose residues in mannans. The physicochemical properties of galactomannan are determined by the Man/Gal ratio in endosperm which can range from 1.0 to 5.6. The Man/Gal ratio of guar galactomannan is 2:1, 1:1 in fenugreek and 20:1 in tobacco. Guar gum content varies considerably from 15.92 to 38.38% in different Indian cultivars^7^.

Whole genome analysis has provided novel opportunities for gene identification and molecular marker development in various model and non-model plants^8^. Mechanisms regulating pod development has been reported in various plants like barrelclover^9^, and peanut^10^. Till date, genomes of legumes namely, soybean, lotus, medicago, pigeon pea, and chickpea have been completely sequenced. Genetic diversity among different clusterbean genotypes has also been explored using commonly used markers such as RAPD, ribosomal DNA (rDNA), simple sequence repeats (SSR), SNP and InDel^11^. Currently, around 78,686 clusterbean sequences including 62,146 unigenes, 16,476 expressed sequence tags (ESTs) and 61 nucleotides are available in the National Centre for Biotechnology Information (NCBI) database. This data has been generated from transcriptome analysis of developing clusterbean seeds and leaves from two clusterbean varieties using Illumina sequencing platform^5,12^. Recently, our group has developed multi-tissue developmental transcriptome database have been generated named as ‘ClustergeneDB’^13^. In addition, chloroplast sequencing of clusterbean *(Cyamopsis tetragonoloba L.)* has also been completed^14^. We have also elucidated novel miRNAs and suggested their role in regulating galactomannan biosynthesis in various tissues of clusterbean^15^. An earlier study has identified and characterized genes related to gum biosynthesis in guar^16^. Recently, we have reported galactomannan biosynthesis pathway genes in two contrasting genotypes (HG365 and HG870) for maturity trait^17^. However, there is no information on spatio-temporal expression of galactomannan biosynthesis related genes and their linked transcription factors (TFs) in contrasting genotypes during different pod development stages. A thorough genomic characterization during different endosperm developmental stages is required which will help in utilizing the molecular resources towards improving the gum content in this crop.

To meet the global demand of the guar seeds/gum, new varieties with higher gum content are urgently needed. For this purpose, availability of complete genomic information including controlling elements like transcription factors (TFs), gene regulatory networks involved in the galactomannan biosynthetic pathway will form a basis. In this regard, the present investigation explored the galactomannan biosynthesis mechanisms at different pod developmental stages in two genotypes contrasting for galactomannan content so as to identify and understand the key genes and TFs involved in the biosynthesis and accumulation of the same in the endosperm.

## Results

### Morpho-physiochemical analysis and galactomannan estimation in RGC-936 and M-83

**Figure 1:**
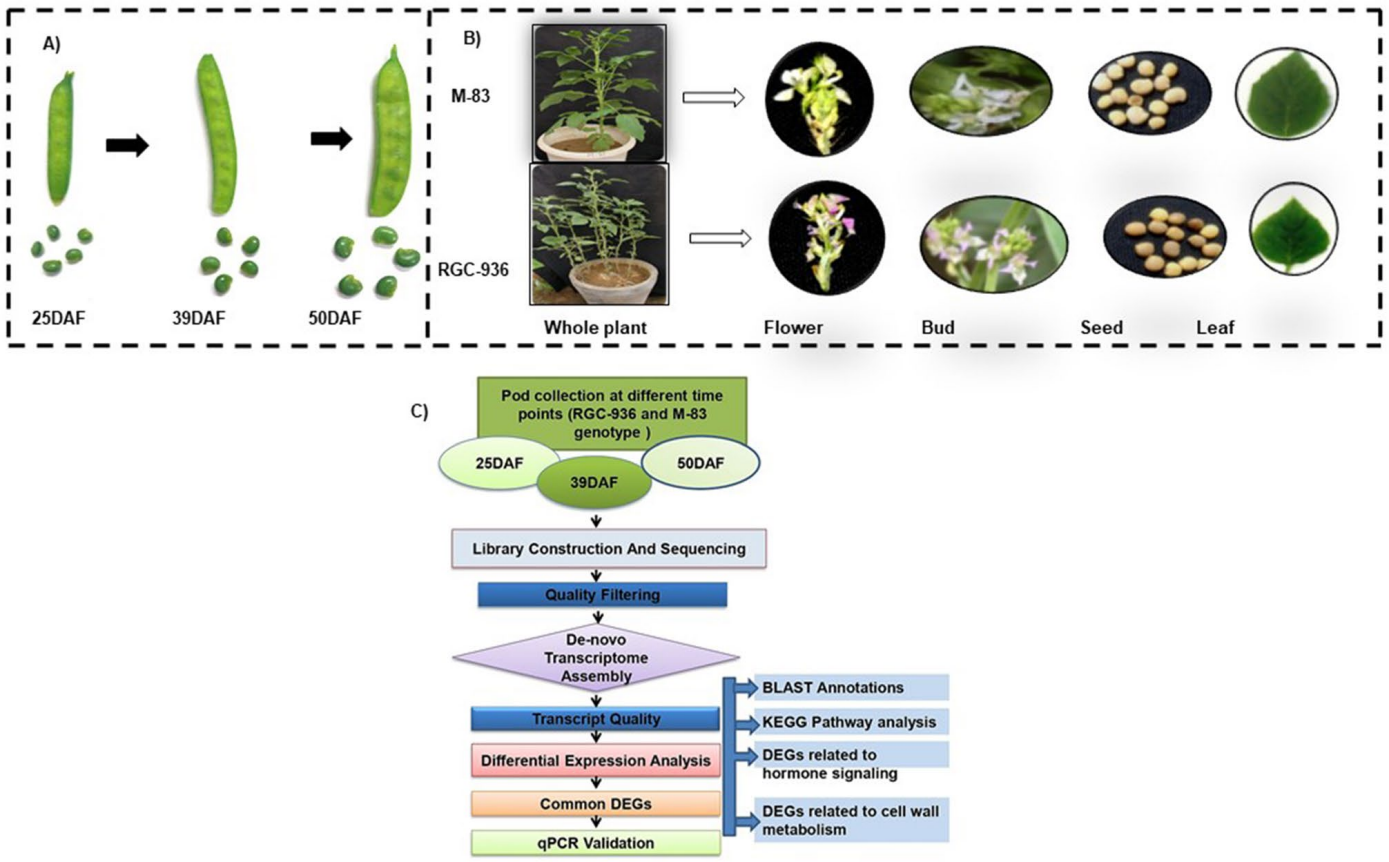
(**A**) Pod development stages (25, 39 and 50 DAF). (**B**) Morphological difference between two genotypes RGC-936 and M-83 and (**C**) Transcriptome analysis pipeline for identification of DEGs genes related to galactomannan biosynthesis in clusterbean.

Morphological observation revealed that RGC-936 has hairy rough leaf surface, purple flower color, 3–4 cluster plant^−1^, and 4–7 pods cluster^−1^, 100 seed weight (2.6 g), small and round seed with taller plant stature. On the contrary, M-83 variety has smooth leaf surface, white flower color, 2–3 cluster plant^−1^, 2–4 pods cluster^−1^, 100 seed weight (2.2 g), big and flat seeds, and shorter plant stature (Fig. 1A,B). Also, M-83 recorded higher chlorophyll content in comparison to RGC-936. Gum content at 39DAF was found to be 24–26% and 14–16% in RGC-936 and M-83 respectively.

### Summary of transcriptome sequencing analysis

For transcriptome analysis, RNA extracts from two genotypes (RGC-936 and M-83) during different pod development stages were subjected to Illumina Hiseq X Ten sequencing, which generated a total of 286.85 Mb raw sequencing reads from 12 RNA libraries. After filtering out low quality reads and adaptor removal, 285.71 Mb clean reads were obtained. A total of 86.05 GB data was obtained from the transcriptome sequencing (150 × 2) bp. Finally, through this high-quality data, 422,998 contigs were assembled with an average length of 201 bp and an N50 value of 1,069 bp (Table 1, Supplementary Table 1A). The sequence reads have been submitted to NCBI-SRA database (Submission ID: SUB5664803, BioProject: PRJNA545776). Differentially expressed genes (DEGs) of the fifteen possible combinations viz. R25-M25, R39-M39, R50-M50, R25-M39, R25-M50, R39-M50, M25-R39, M25-R50, M39-R50, R25-R39, R25-R50, R39-R50, M25-M39, M25-M50, M39-M50 were filtered at log2fold change and significantly DEGs (*p* ≤ 0.01) were found to be 1489, 4417, 1198, 3180, 1958, 5044, 2778, 1710, 3202, 3766, 1964, 4849, 1997, 2450 and 4192 unigenes, respectively. Transcriptome analysis pipeline for identification of DEGs related to galactomannan biosynthesis in clusterbean has been shown in Fig. 1C.

**Table 1:** Statistical analysis of non-redundant assembly and average mapping percentage (with two biological replicates) at three pod developmental stages (25DAF, 39DAF and 50DAF) in two clusterbean genotypes RGC-936 and M-83.

(A) Non-redundant assembly statistics						
Raw reads	Clean reads	N50	Number of sequence	Total length	Minimum contig length	Largest contig length
286,853,239	285,714,375	1069	422,998	238,400,658	201	15,133

(B) Mapping statistics						
Sample name/2 replicates	Clean reads	Average mapping % (with two biological replicates)				
R25	52,136,303	99.18				
M25	54,676,755	99.11				
R39	43,655,782	97.65				
M39	45,076,268	99.56				
R50	41,863,772	99.43				
M50	48,305,500	99.42				

### GO classification and KEGG enrichment analysis of DEGs

GO annotation of the DEGs from the 15 pairwise comparisons (mentioned above) were used to classify genes into three main GO categories (present in the gene ontology annotations) based on total gene count and their role in different functional processes: information storage and processing category, metabolic and cellular function. KEGG enrichment analysis allowed mapping of DEGs to top 20 pathways. At 25DAF (early development), highly expressed genes belonged to metabolic pathways, ribosome, oxidative phosphorylation, photosynthesis and secondary metabolite biosynthesis. At 39DAF (mid development), higher expression was observed for genes involved in metabolic pathways, glutathione biosynthesis, secondary metabolite biosynthesis and cysteine methionine metabolism; while late developmental stage i.e., 50DAF showed higher expression of genes involved in metabolic pathways, secondary metabolite biosynthesis, antibiotic biosynthesis, carbon metabolism, oxidative phosphorylation, amino acid biosynthesis, and carboxy acid metabolism. Thus, genes involved in metabolic pathways and secondary metabolite biosynthesis were common to all the three seed developmental stages, while oxidative phosphorylation genes were prominent at 25 and 50 DAF.

### Identification and comparison of number of DEGs potentially involved in galactomannan biosynthesis at three pod developmental stages in clusterbean

Total 209,525, 375,595 and 255,401 unigenes were found at three-time points i.e., R25-M25, R39-M39 and R50-M50 with a higher number of genes at 39DAF followed by 50DAF. Differential gene expression analysis showed a total of 2384 DEGs (1131 up-regulated and 1253 down-regulated), 4780 DEGs (3934 up-regulated and 846 down-regulated) and 1792 DEGs (1238 up-regulated and 554 down-regulated) between R25-M25, R39-M39 and R50-M50 groups respectively. Results showed that highest number of DEGs were expressed during the middle stage of pod development i.e., 39DAF, followed by 25 DAF and 50 DAF (Supplementary Table 1B).

**Figure 2:**
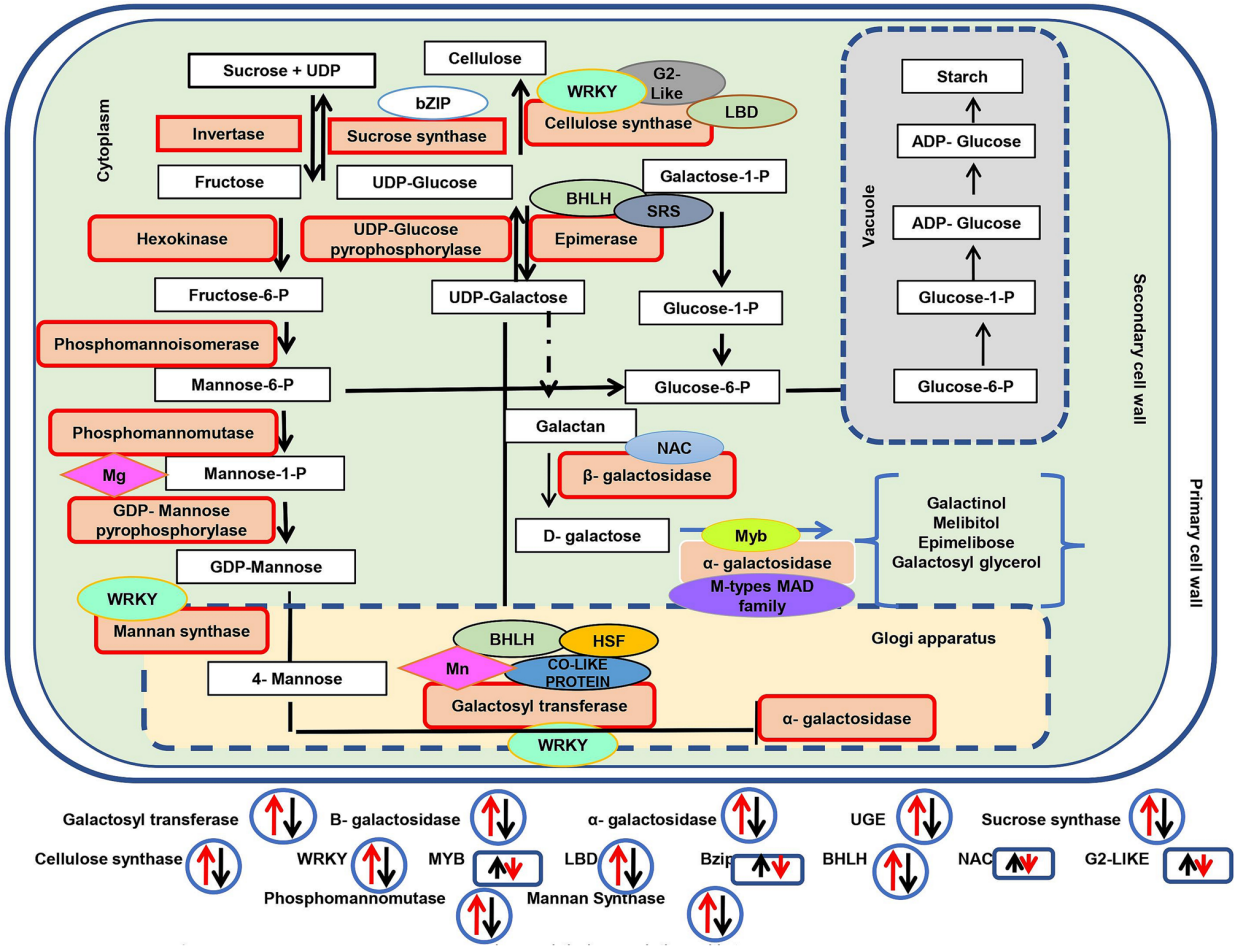
Galactomannan biosynthesis pathway and galactomannan related gene expression and their correlation with transcription factor in clusterbean. Red arrow denotes relative expression in RGC-936 and black arrow denotes relative expression in M-83 genotype at 39DAF pod developmental stage (using Microsoft Powerpoint based on transcriptome data).

To elucidate the potential pathways involved in galactomannan biosynthesis, we used annotated galactomannan pathway genes in soybean (*Glycine max*) as a reference sequence. (Supplementary Table 2). Systematic illustration of galactomannan metabolism pathway in clusterbean has been shown in Fig. 2. These included genes encoding for invertase, sucrose synthase, cellulose synthase, GDP- mannose pyrophosphorylase, phoshphomannomutase, phosphomannoisomerase, UDP-galactose-4-epimerase, mannan synthase, glycosyltransferase, galactosyltransferase, endo-β-1,4-mannanase and β mannosidase. Most of the predicted gum related genes showed highest expression in 39DAF followed by 50DAF and least expression in 25DAF. A total of 53, 51, and 43 unigenes, related to sucrose synthase were identified at 25DAF, 39DAF, and 50DAF respectively in clusterbean.

No ESTs related to Phosphomannoisomerase (EC 5.3.1.8) were detected in any of the developmental stages studied. However, nine unigenes which resembled phosphomannomutase (EC 5.4.2.8) (catalyses the reversible conversion of D-mannose 6-phosphate to α-D-mannose 1-phosphate) were identified at 25DAF, while eleven such unigenes were found at 39DAF and nine unigenes at 50DAF. GDP-D-mannose and UDP-D-galactose, the direct precursors for galactomannan biosynthesis are synthesized by enzyme GDP mannose phosphorylase (EC 2.7.7.22) and UDP-galactose 4-epimerase (EC 5.1.3.2) respectively. A total of 27, 33, and 26 unigenes of UDP-glucose-4-epimerase were detected at 25DAF, 39DAF, and 50DAF respectively, while 9 unigenes were observed for GDP-mannose transporter at 25DAF and 21 unigenes at both 39DAF and 50DAF stages.

For endo-β-mannanase, a total of 21, 24, and 23 unigenes, while for cellulose synthase like genes, 165, 161 and 156 unigenes were identified at 25DAF, 39DAF, and 50DAF respectively.

For Mannan synthase (ManS), a key enzyme of galactomannan metabolism, 6 unigenes were found at 25DAF while 2 unigenes were found at both 39DAF and 50DAF. Further, we identified a total of 0, 36 and 39 unigenes related to glycosyltransferase, 135, 136 and 127 unigenes related to α-glucosyltransferase and β-glucosyltransferase, 44, 54 and 53 unigenes related to α-galactosidase, and 137, 145 and 146 β-galactosidase unigenes at 25DAF, 39DAF and 50DAF respectively. Details have been provided in Supplementary Table 3. Out of these unigenes, 1367 (at 25DAF), 2715 (at 39DAF) and 1065 (at 50DAF) unigenes were found to be differentially expressed between two genotypes (Fig. 4B).

### Co-expression of gum synthesis genes with identified TFs

**Figure 3:**
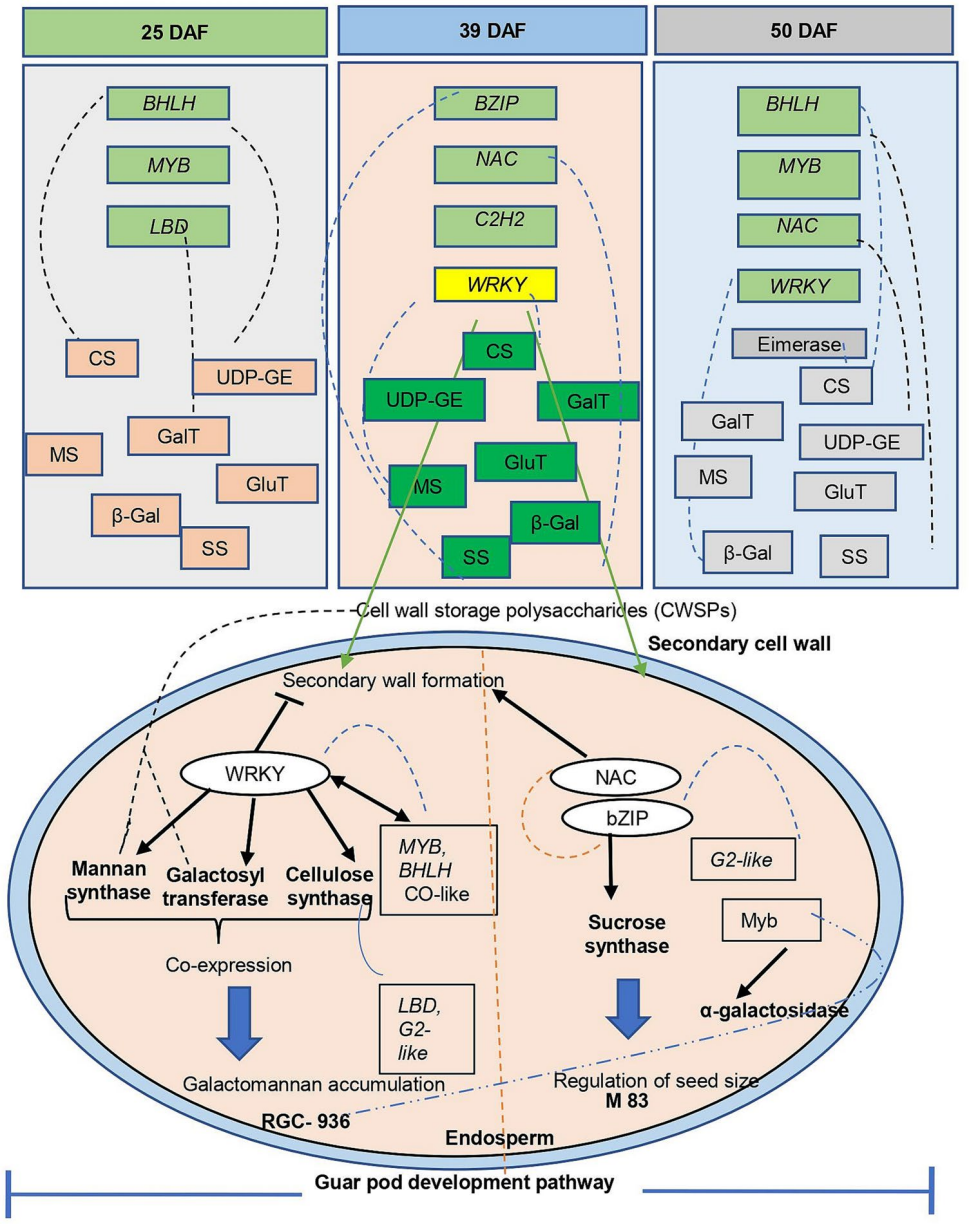
Model for *WRKY* and other TF regulating galactomannan biosynthesis in clusterbean: *WRKY* targets 3 genes (Mannan synthase, galactosyl transferase and cellulose synthase) related to galactomannan metabolism. Bold arrow shows enzyme targeted by TF, while dotted blue arrow shows indirect involvement and red arrow shows synergistic role (using Microsoft Powerpoint based on transcriptome data).

Seed development is a highly coordinated process controlled by various TFs. A total of 15 combinations among three developmental stages (25, 39 and 50DAF) were analyzed for TFs/genes related to gum synthesis. Total numbers of annotated genes were found to be 50,275, 49,376, 49,656, 49,376, 49,686, 48,287, 50,095, 49,686, 48,075, 49,999, 49,475, 49,194, 49,622, 49,968, and 48,287 in 15 combinations viz. R25-M25, R39-M39, R50-M50, R25-M39, R25-M50, R39-M50, M25-R39, M25-R50, M39-R50, R25-R39, R25-R50, R39-R50, M25-M39, M25-M50, M39-M50 respectively. However, after filtering the fold change value (p ≤ 0.01 and p_adj_ ≤ 0.01), the highest number of genes were identified in M39-M50 (1566) followed by M39-R50 (1218). Stage-specific TFs were also identified such as *BHLH* family protein, *MYB* related family protein, *LBD, BZIP, NAC,* and *C2H2.* Members of *the bZIP, NAC, WRKY* and *C2H2* families were specifically found to be highly expressed at 39DAF in RGC-936. A model for *WRKY* and other TFs regulating galactomannan biosynthesis in clusterbean is illustrated in Fig. 3.

Some of the *in-silico* TFs that were found to be co-expressed with the genes regulating galactomannan biosynthesis were validated using real time PCR. Cellulose synthase expression was found to be highly correlated with *LBD, G2-Like* and *WRKY* TFs. β-galactose was found to be co-expressed with *NAC* TF; epimerase with *BHLH* TF; mannan synthase with *WRKY*; galactosyltransferase with *BHLH, WRKY* TF; α galactosyltransferase with *BHLH* and *WRKY* TF; and sucrose synthase with *bZIP* TF (Fig. 2).

### Expression profiling of genes involved in galactomannan biosynthesis using the heat map

**Figure 4:**
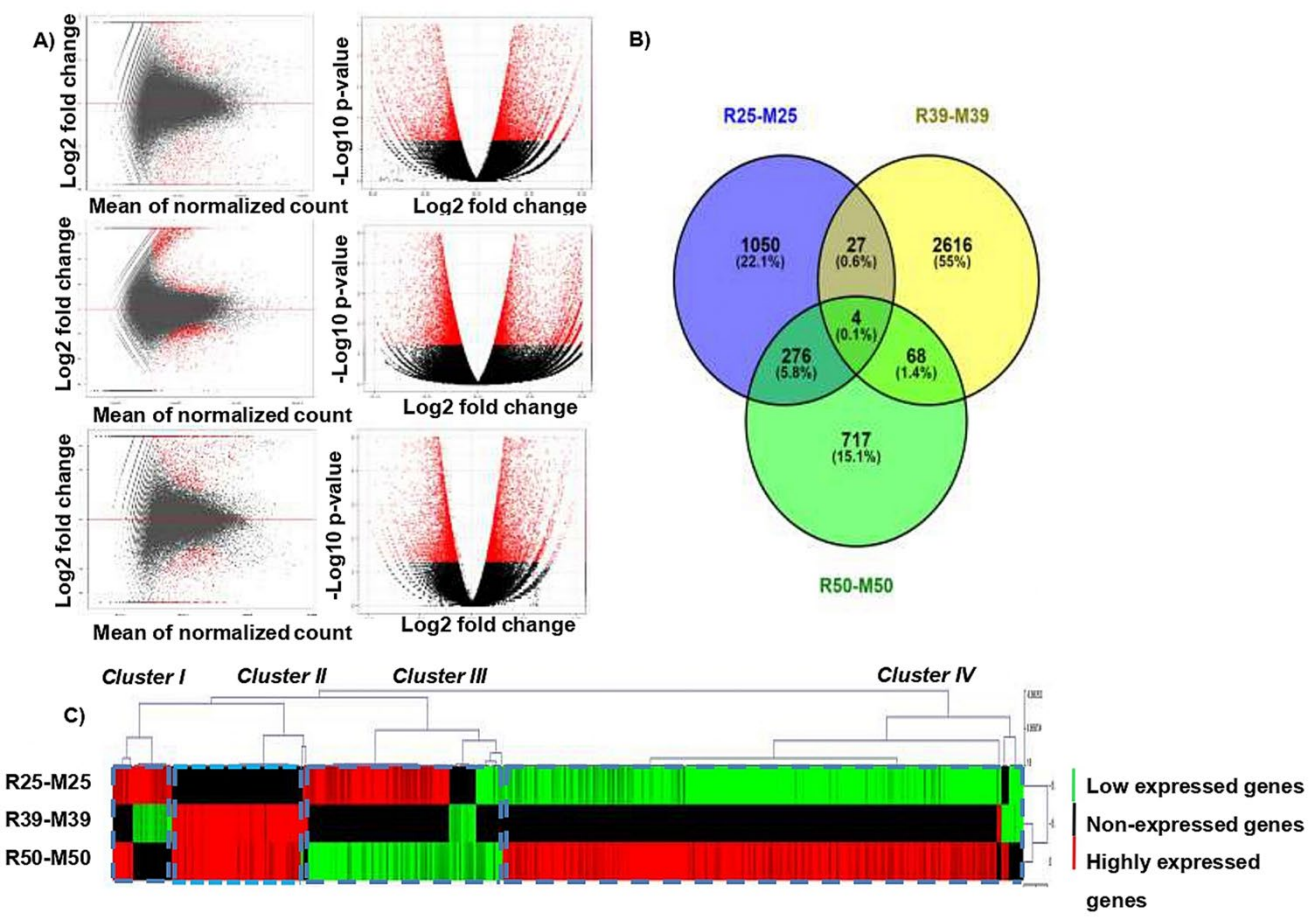
Differential expressed genes at three pod developmental stages (25DAF, 39DAF and 50DAF) between two contrasting genotypes RGC-936 and M-83 in clusterbean as shown by (**A**) Volcano plot (using R package), where red color dots represent expressed genes and black color dots represent non expressed genes, (**B**) Venn diagram (using Venny software) and (**C**) Heat map diagram (using R package).

MeV software was used for Hierarchical Clustering Analysis (HCA) of DEGs according to their transcription profile. The HCA was performed to see the distribution of DEGs present in the transcriptome data at three pod development stages. Interestingly, we found that the highly expressed genes (indicated by red color) showing annotation of the important genes involved in galactomannan biosynthesis are grouped in cluster II mainly at mid and late development stages in RGC-936 (Fig. 4C). No expression was observed at 25DAF in both the genotypes.

### Real time PCR validation and spatiotemporal expression profiles of genes potentially involved in galactomannan biosynthesis

**Figure 5:**
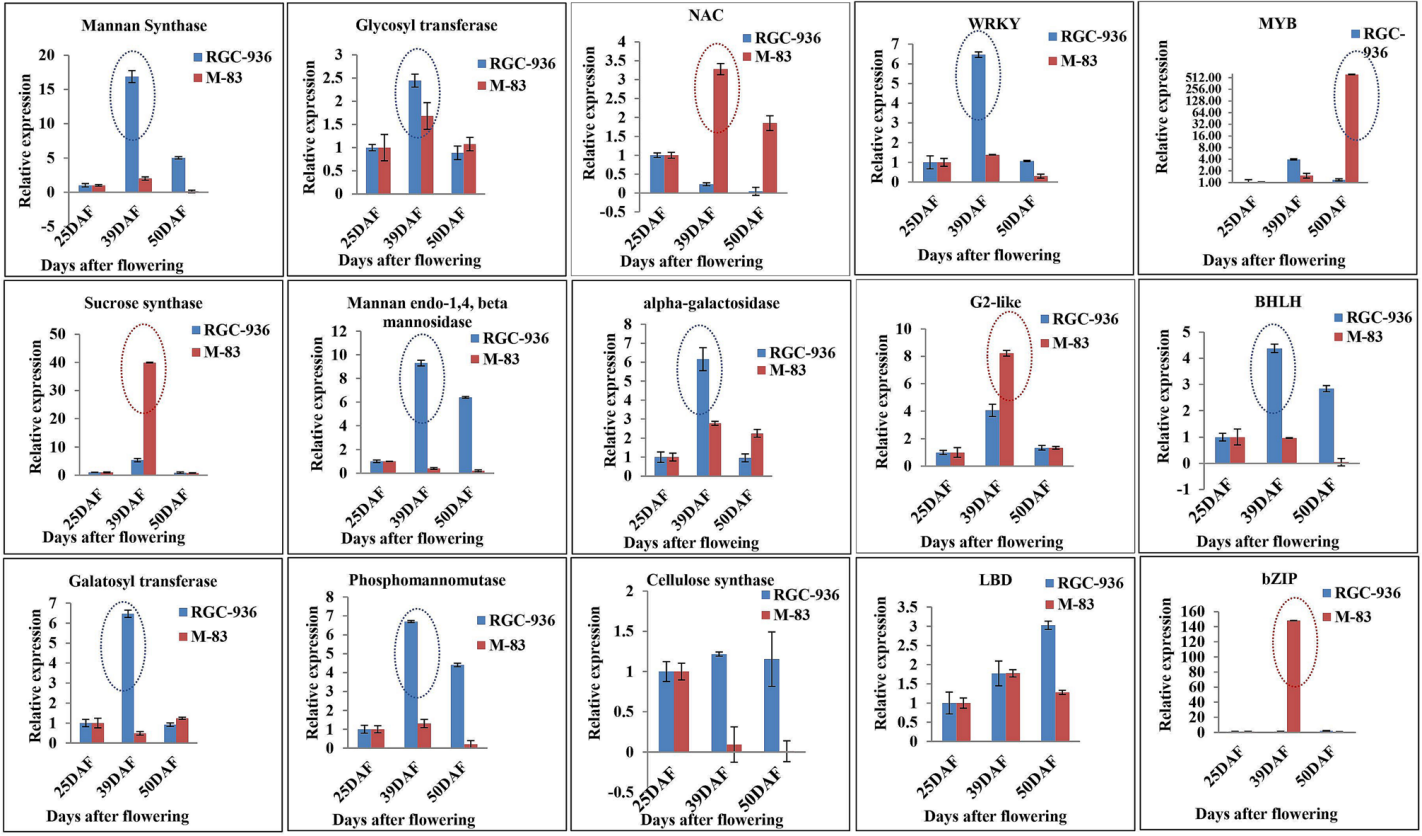
The expression levels and patterns of 15 selected unigenes associated with galactomannan biosynthesis in RGC-936 and M-83 at different pod developmental stages 25DAF, 39DAF and 50DAF in clusterbean were confirmed by qRT-PCR (Using Microsoft Excel).

A total of six RNA samples with two biological replicates each were isolated from pod tissues at different developmental stages (25DAF, 39DAF and 50DAF) from RGC-936 and M-83. The expression profile of the enzymes directly related to galactomannan biosynthesis was validated by RT-PCR at 39DAF in RGC-936 which correlated well with *in-silico* data, except for sucrose synthase which showed higher expression in M-83 at 39DAF. The relative expression of α-galactosidase, mannan synthase, glycosyltransferase, cellulose synthase, Mannan endo 1,4 β mannosidase, and phosphomannomutase were found to be significantly higher at 39DAF and 50DAF in RGC-936 as compared to M-83 (Fig. 5). For sucrose synthase, relative expression was found to be 5.4-fold at 39DAF and 0.9-fold at 50DAF in RGC-936, while it was 39.9-fold at 39DAF and 0.8-fold at 50DAF in M-83 (Fig. 5).

### Real time PCR validation and spatiotemporal expression profiles of TFs potentially involved in galactomannan biosynthesis

The relative expression level of TFs including *WRKY, BHLH* was found to be higher at 39DAF in RGC-936, while expression of *NAC*, *G2-Like*, *bZIP* was higher in M-83 at 39DAF. *MYB* relative expression was found to be 3.9-fold at 39DAF and 1.2-fold at 50DAF in RGC-936, while it was 1.5-fold at 39DAF and 621.7-fold at 50DAF in M-83. Expression of *LBD, G2 Like,* and *BHLH* was found to be higher at 39 DAF in both the genotypes. *bZIP* relative expression was found to be 1.1-fold at 39DAF and 1.9-fold at 50DAF in RGC-936, while 148.2-fold at 39DAF and 0.7-fold at 50DAF in M-83 (Fig. 5).

### Electron microscopy analysis of seeds of RGC-936 and M-83 genotypes

**Figure 6:**
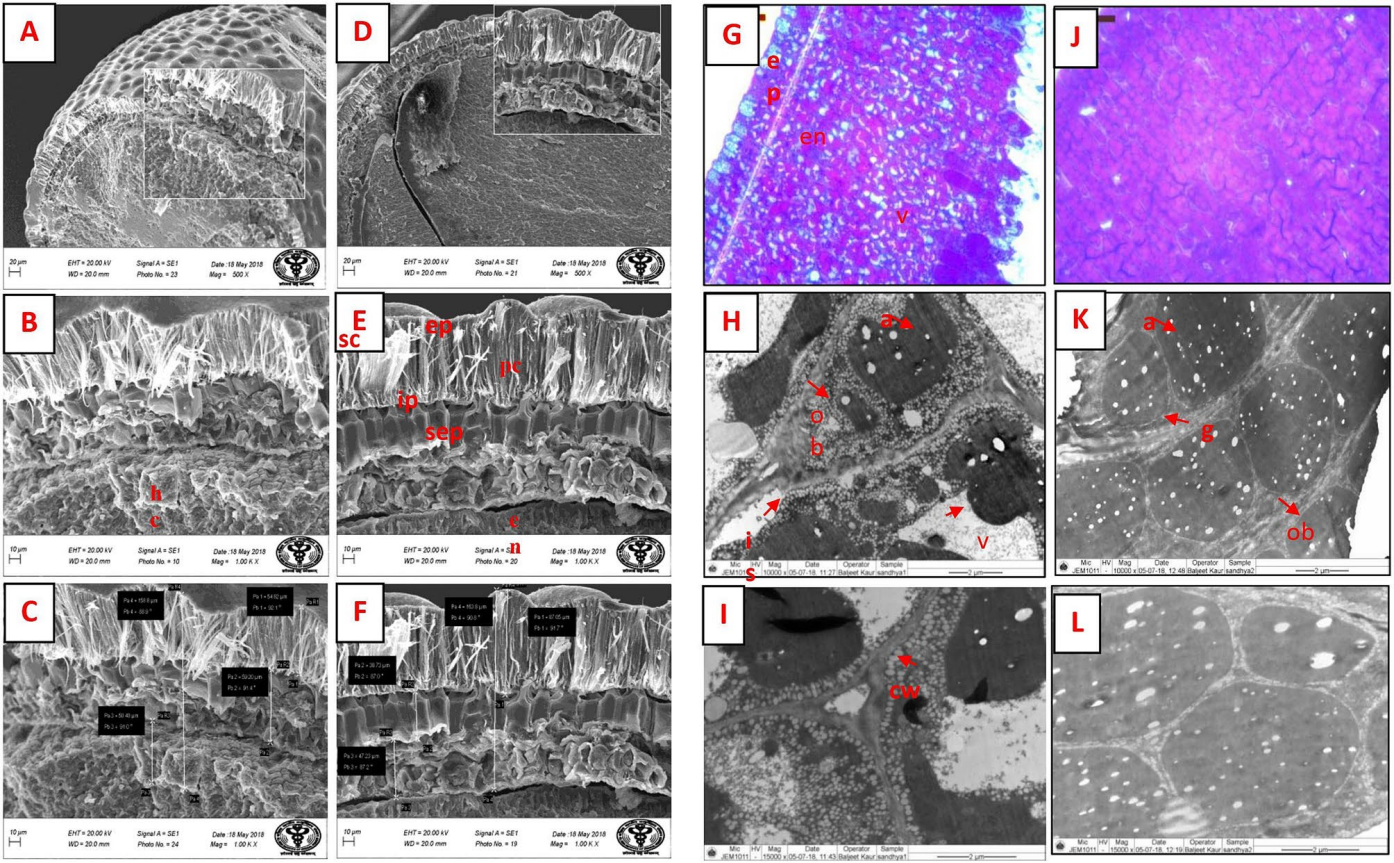
Comparative electron micrograph and light microscopic cross sectioned seed images of M-83 and RGC-936 genotypes. (**A**–**C**) represent scanning electron microscope of M-83. (**D**–**F**) represent cross sectioned image of RGC-936. G represent light microscopic images of M-83 stained with toluidine blue while (**H**,**I**) represent Transmission electron micrograph of M-83 seeds. (**J**) represent light microscopic images of RGC-936 stained with toluidine blue while (**K**,**L**) represent Transmission electron micrograph of RGC-936 seeds. *Hg* hourglass cells, *pc* palisade cells, *sep* sub epidermal pillar cells, *en* endosperm, *ep* epidermis, *sc* seed coat, *g* globoids, *v* vacuole, *a* alurone, *ob* oil bodies, *is* intracellular spaces, *cw* cell wall.

Scanning electron microscopy (SEM) micrographs of the mature seeds showed larger cuticle structure with more space in RGC-936 as compared to smaller cuticles in M-83. Further, the mature seed coat of RGC-936 (Fig. 6) in surface view showed epidermal as well as hypodermal wall thickenings underneath, a well-defined sub-epidermal layer, and a larger endosperm than M-83. RGC-936 had thicker seed coat (163.8 µm), rich in protein bodies with compact and uniform distribution of aleuroplast cells, and contained a greater number of globoids as compared to M-83 which had moderately thick (158.8 µm) seed coat, scattered aleuroplast, large number of oil bodies and intracellular spaces due to the larger vacuole. The starch content was lower in M-83 seeds with a greater number of oil bodies as compared to RGC-936.

## Discussion

Characterization of genes and their functions during seed/pod development is an important step towards the understanding of the regulation of biosynthesis of important macromolecules. Whole transcriptome analysis provides a comprehensive understanding of the complex interplay of the various pathways involved in these biosynthetic/ developmental processes. Therefore, to gain new insight into the galactomannan biosynthesis in clusterbean, a comparative transcriptome study was carried out. The primary objectives of our study were (a) comparison of the transcriptome at three pod development stages in two contrasting guar genotypes, (b) identification of DEGs and their co-expression with TFs involved in galactomannan biosynthesis, and (c) biochemical and ultra-structural characterization of seeds in these two genotypes.

Out of the total 5147 DEGs between two genotypes at three time points, a major alteration in gene expression was observed at 39DAF, particularly in RGC-936. This seems true for the genes which are directly involved in galactomannan biosynthesis (Fig. 2). This pattern of expression of genes involved in galactomannan biosynthesis observed in our study is consistent with previous reports showing the highest expression of genes at 35DAF followed by decrease in later stages^5^. It seems that a stage-specific posttranscriptional control regulates galactomannan gene expression in clusterbean embryos. Annotation of transcripts led to identification of a total of 15 enzymes directly or indirectly involved in galactomannan biosynthesis, of which phosphomannomutase (count 11), mannan endo-1,4-β-mannosidase (count 24), α-galactosidase (count 54), β-galactosidase (count 145), UDP-Glycosyltransferase (count 36), UDP-glucose/galactose 4 epimerase (count 36), α-1,3-glucosyltransferase (count 9), β-glucosidae (count 211) and cellulose synthase (count 165) were found in RGC-936, while sucrose synthase (count 53), and glucosyltransferase (count 135) were found in M-83 at 39DAF (Supplementary Table 3). In our previous study^15^, we observed tissue-specific galactomannan genes expression and their associated mi-RNAs. A total of 38 unigenes coding for sucrose synthase and 67 unigenes coding mannosidase (targeting Ct-mi482), 29 unigenes coding for epimerase (targeting Ct-miR319), and 175 unigenes coding for cellulose synthase targeting two miRNAs Ct-miR3130 (known) and Ct-miR3135 (novel) were identified^15^.

Comparative in-silico transcriptome data and quantitative PCR expression analysis revealed significant increase in transcript levels of galactomannan biosynthesis unigenes encoding mannan synthase, galactosyltransferase, UGE, cellulose synthase, phosphomannomutase in RGC-936 as compared to M-83, however the transcript levels of the unigene encoding sucrose synthase was significantly increased in M-83 (Fig. 5). At 39DAF, when galactomannan content is at its peak, the TFs like *WRKY*, *BHLH, MYB*, *LBD* showed significantly higher expression in RGC-936 as compared to M-83 indicating an important role of these TFs (especially *WRKY* protein family *(WRKY 20)* and *BHLH*) in gum accumulation at 39DAF.

TFs mediate gene expression by binding to specific DNA elements and motifs. We also observed the co-expression of TFs and genes involved in galactomannan biosynthesis. Mannan synthase, the core enzyme of galactomannan biosynthesis that makes the β-1, 4-mannan backbone of galactomannan, in guar seed endosperm walls was found to be co-expressed with *WRKY* at 39DAF in RGC-936. *WRKY* targets many genes linked to polysaccharide metabolism and also acts as negative regulator for secondary wall formation in *Dendrobium Officinale*^18^. Galactosyltransferase which substitutes galactosyl residues from UDP-galactose to the mannan backbone was observed to be co-expressed with *WRKY, MYB, BHLH* TFs. Earlier studies have also hinted at synergistic role of *MYB, BHLH* and others during galactomannan biosynthesis^15,19,20^. These two genes (mannan synthase and galactosyltransferase) work in concert and their transcription peak correlates with cell wall storage polysaccharides (CWSPs) in seed endosperm^21,22^. In our study, both genes were found to be co-expressed with *WRKY*, highlighting their synergistic role in galactomannan biosynthesis.

The third important enzyme, α-galactosidase which hydrolyses the terminal galactosyl residue and thus control galactose substitution^6^ was found to be co-expressed with *MYB* TF at 39DAF in RGC 936 highlighting their synergistic effect. Sucrose synthase transcripts have also been observed to be elevated during seed development in *Arabidopsis thaliana, Citrus unshiu, and Cyamopsis tetragonoloba*^23^*.* In present study, expression of sucrose synthase (EC 2.4.1.13) correlated positively with *NAC* TF at 39DAF. *NAC* has been reported to participate in many developmental processes^24^ including SCW formation^25^ and is also involved in regulation of seed size. This might be the explanation for larger seed size of M-83. Surprisingly, the expression level of *bZIP* family TFs was found to be significantly higher (approximately 140-fold) in M-83 as compared to RGC-936, and expressed only at the mid-development stage. It is notable that *bZIP*, a direct regulator of sucrose synthase also acts synergistically with *NAC* and *G2-Like* TF. *bZIP* TF has been reported to be involved in various sucrose signaling processes^26^. Transcription of *ATB2/AtbZIP11* is stimulated by sugars and light, while translationally repressed by higher sucrose concentrations^27^. On the contrary, expression of*TF bZIP1* is repressed by sugars and upregulated by energy deprivation at transcriptional as well as post-transcriptional levels^28^. Cellulose Synthase-Like (CSL) proteins belong to a group of enzymes that synthesize β-1, 4-linked polysaccharide backbones of mannans. In our study, cellulose synthase seems to be regulated by *LBD*, *G2-Like* and *WRKY* TF family.

Three enzymes involved in galactomannan biosynthesis viz., mannan synthase, galactosyltransferase and cellulose synthase were specifically observed to be associated with *WRKY* TF family at 39DAF hinting at its probable role in pod development, and regulation of galactomannan biosynthesis in clusterbean (Supplementary Fig. 1), though it needs further functional validation.

Dynamic comparison of mature seeds of both the genotypes showed significant correlation between starch/oil and protein accumulation as indicated in electron microscopy and histochemical studies. Similar reports are also available in legume seeds of *Glycine max; Cicer arietinum; Lupinus luteus; Pisum sativum; Phaseolus vulgaris; Lotus japonicas*^29–33^ and other crops^34^. Histological studies using toluidine dye showed large vacuoles in intracellular spaces of M-83, while structure of RGC-936 was compact (Fig. 6). Thickness of seed coat of RGC-936 (163.8 µm) was found to be larger as compared to M-83 (158.8 µm). The inner layer of the seed coat known as aleurone is a living layer that protects the embryo during germination by mobilizing its energy reserves^35,36^. RGC-936 was observed to be rich in protein bodies with compact and uniform distribution of aleuroplast cell, while M-83 showed scattered aleuroplast, large size and number of oil bodies. The presence of protein bodies^2^ has also been reported in embryonic cells of many other legume seeds like *Arachis hypogaea, Medicago sativa, Phaseolus lunatus, Pisum sativum, Vigna unguiculate, Lupinus albus, L. angustifolius and L. luteus* as well as seeds of other species. In silico analysis of seed storage protein showed approximately 16% transcripts coding for oleosin gene next to legumin. Less oil usually corresponds to high starch content, but not essentially^33,37^. M-83 with a greater number of oil bodies (experimentally seen in TEM images) showed lower starch content as compared to RGC-936 where the number of oil bodies were comparatively less. The negative correlation of oil body and starch content has also been reported in crops like *Glycine max* (20% oil and 2% protein)^29^, while reverse has been observed in *Cicer arientinum* (6% oil and 44% protein)^30,32^ and in *Vicia faba* (3% oil and 45% Protein)^29^. Biochemical analysis using K-GALM Kit for galactomannan estimation showed a significant difference in gum content between two genotypes. RGC-936 (24–26%) showed higher accumulation of gum content as compared to M-83 (12–14%). Galactomannan content varies between 26–32% among mature seeds of different guar genotypes^38^. Therefore, lower oil bodies and high oleosin in RGC 936 suggests high oil content/starch/gum content in comparison to M-83. Further, higher expression of genes coding for olesin in RGC 936 support the evidence of low oil body and high oil content in this genotype.

Our findings shed light on the important TFs regulating galactomannan biosynthesis genes at different pod development stages. Real time validation confirms the synergistic/antagonistic expression of TFs with targeted gene. Not only transcript expression, but biochemical assays and electron microscopy study results also revealed significant differences in galactomannan accumulation and distribution of oil bodies/protein between the two genotypes. *WRKY* TF family might be a key regulator of galactomannan accumulation, but it needs further validation, and *bZIP* is straightway regulator of sucrose synthase in clusterbean.

## Conclusion

Overall, RNA-Seq analysis during pod development in two contrasting genotypes has enabled us to carry out a global investigation of gene expression at three-time points in developing clusterbean and has aided in generating an inclusive picture of transcriptional events that occur differentially between galactomannan rich (RGC-936) and galactomannan poor (M-83) clusterbean genotypes. The functional ontologies at three developmental stages showed high expression of large number of genes related to biological processes predominantly at 39DAF. Co-expression of *WRKY* with key genes of galactomannan pathways pinpoints *WRKY* as an important molecular component in the galactomannan metabolism in clusterbean. These results provide a basis for further characterizing the role of *WRKY* genes to understand the downstream regulation network during pod development in cluster bean.

## Methods

### Sample collection and growth conditions

The seeds of clusterbean genotypes (RGC-936 and M-83) were obtained from Central Arid Zone Research Institute, Jodhpur, India. The seeds were germinated in the pots (size 8 × 8") during kharif season at Net House, NIPB, IARI, New Delhi. Total five seeds per pot were sown and after germination, plants were thinned to maintain only 2 plants per pot. The plants were irrigated every alternate day. The pod tissues were collected from each plant mentioned above at different developmental stages viz. 25DAF, 39DAF and 50DAF (Fig. 1A) from first flowering stage and immediately stored at − 80 °C for further RNA isolation and comparative transcriptome analysis.

### Morpho-physiochemical analysis

Under morphological parameters, the traits like leaf surface, flower colour, cluster plant^−1^, pod cluster^1^, 100 seed weight, plant height, seed shape, flowering time and chlorophyll content were recorded along with physiological and biochemical parameters like chlorophyll content, and galactomannan content respectively. Galactomannan content at mature seed stage of two variety of clusterbean was estimated using K-GALM 03/13 kit as per the manufacturer’s protocol (Megazyme, USA).

### Microscopic study of mature seeds: light microscopy, SEM and TEM

For light microscopy, the samples were fixed in FAA (5% acetic acid, 4% formalin in 50% ethanol), dehydrated in serially increasing strengths of ethanol, and embedded. The embedded material was sectioned with a Reicher-Jung 1150/Autocut rotary microtome and stained in Toluidine Blue^39^. The sections were observed under an Olympus BX61 light microscope.

For SEM, samples (seeds) were cut into small pieces and put into a fixative solution consisting of 2.5% glutaraldehyde and 2% paraformaldehyde in 0.1 M sodium phosphate buffer (pH 7.3)^40^. Samples were fixed for 12 h at 4 °C. Post fixation, samples were washed in buffer and again fixed in 1% OsO_4_ for 2 h at 40 °C. Thereafter, dehydration of samples was performed serially in an ascending grade of acetone, dried at critical point using Critical point dryer, Polaron, followed by mounting on aluminium stubs. Samples were sputter coated (SCD 050 Super Cool Sputter System; Baltec Technology, Liechtenstein) with colloidal gold and observed under an Evo 18 scanning electron microscope (Carl Zeiss). Images were captured using Smart SEM software at Magnification 1.00 K X. For Transmission Electron Microscopy (TEM), the samples were dehydrated in acetone as done in case of SEM, following which they were infiltrated and embedded in araldite CY 212 (TAAB, UK) and polymerized. These preparations (0.5 µm thick) were cut using an ultra-microtome (Leica Ultracut UC7, Austria). Sample sections were stained with uranyl acetate and alkaline lead citrate for 10 min each^41^. Post-staining, samples were washed gently with distilled water and observed (magnification 10,000 to 15000×) under a Tecnai G2-20 S-Twin high-resolution transmission electron microscope (Fei, The Netherlands) at SAIF-New Delhi, All India Institute of Medical Sciences (New Delhi). Images were captured digitally using Digital Micrograph software.

### RNA Library preparation, sequencing and assembly

Total RNA was extracted from pod tissues of RGC-936 and M-83 at three developmental stages (25DAF, 39DAF and 59DAF) in two biological replicates using Sigma Aldrich RNA extraction kit and RNA integrity was checked using Bioanalyzer (2100 Agilent Technologies). Total twelve RNA-seq libraries were made using TrueSeq RNA Sample preparation kit V2 (Illumina Inc., USA) and sequenced on HiSeq2000 (Illumina, USA). “NGS QC toolkit” was used to filter the raw reads (150 bp × 2) of all the stages^42^. Phred score Q ≤ 20 were removed. Trim galore (version 0.4.1) was used to trim the adapter sequence and final reads were filtered for quality using FastQC (version 0.11.5, http://www.bioinformatics.babraham.ac.uk/projects/fastqc/) (Table 1). The QC filtered reads were merged together and Trinity assembler with default parameters were used to reassembled. De-novo assembly was done using software trinity (version 2.4.0) with default parameters. Assembled fasta files were clustered to remove redundancy using CD-hit (version 4.6) at 90% sequence similarity. Genome Indexing for non-redundant assembled genome was done using reference-based assembler bwa (Version 0.7.5, http://bio-bwa.sourceforge.net/). Replicates were combined and each group (R25, R39, R50 and M25, M39 and M50) was mapped to non-redundant assembled genome using BWA-MEM algorithm which is a faster and more accurate method (Table 1). TrinityStats.pl script was employed to calculate the statistics of the assembled transcripts. QC filtered reads of each of the samples were assembled via a Bowtie2 aligner and each transcript were accurately quantified with RSEM tool. Differential analysis of the fifteen possible combinations R25-M25, R39-M39, R50-M50, R25-M39, R25-M50, R39-M50, M25-R39, M25-R50, M39-R50, R25-R39, R25-R50, R39-R50, M25-M39, M25-M50, M39-M50 was done using DESeq and R package with a minimum cutoff of twofold change on log2 scale. R based packages (Heat map, Spearman correlation matrix, and cluster analysis) were used for analysis of differentially expressed transcripts. The transcripts were annotated using Fast Annotator online server tool with default parameters^43^. Volcano Plot was drawn using R-script (Fig. 4A).

### Functional annotation, KEGG, transcription factor and GO identification and their classification

Comparative transcriptomic analysis among the three developmental stages was performed using log2-scale intensity value for differential expression analysis. Gene sets (filtered at log2FC and *p* ≤ 0.05) in each combination were mapped against Plant NR database (NCBI non-redundant sequence database) by using an E-value cut-off of 10^–5^ (E-value < 0.00001) using BLASTX. Annotation of the hits obtained was further aligned by BLASTX using Uniprot database, and KEGG database proteins with functional annotations retrieved with the highest sequence similarity for given unigene. Genes were also mapped to Plant TFdatabase (version 4.0) and COG database. Gene Ontology results were plotted using WEGO for visualization and annotation of gene (Web Gene Ontology Annotation Plot http://wego.genomics.org.cn/cgi-bin/wego/index.pl) for 15 possible combinations (Supplementary Table 4 & Table S6).

### Differentially expressed genes analysis

Differential gene expression analysis of the fifteen possible combinations R-25-M25, R-25-M39, R25-M50, R39-M-25, R39-M39, R39-M52, R52-M25, R52-M39,R52-M52, R25-R39, R25-R52, R39-R52, M25-M39, M39-M52, and M25-M52 (R Denotes RGC-936 and M, M-83) was done using DESeq an R package. DEGswere filtered at log2 fold change and significant genes were expressed at *p* ≤ 01.

### Total RNA isolation, cDNA synthesis and qRT- PCR analyses

Total 33 sets of oligos from genes (including enzymes and TFs) related to galactomannan biosynthesis pathway were selected based on *p* ≤ 0.01for wet lab validation and designed by using online tool PrimerQuest (https://sg.idtdna.com/Primerquest/Home/Index) from Integrated DNA technologies. Out of which only 15 worked well in the semi-RT result (Supplementary Table 5). Total RNA was isolated in triplicate followed by purification step using Plant Total RNA isolation Kit (Sigma Aldrich, USA) according to the manufacturer’s instruction. The quality and quantity of isolated RNA was assessed using gel electrophoresis on 1% formaldehyde agarose gel and UV–visible Varian spectrophotometer, model Cary 100 by measuring the absorbance at 260 nm respectively. The first-strand cDNA synthesis was done using Reverse Transcriptase (Thermo Scientific) as per the manufacturer’s protocol and diluted to 1:10 ratio for real-time expression studies. To validate the results obtained from the transcriptome data, the relative expression levels of 15 selected genes were recorded by qRT-PCR, using SYBR Premix Ex TaqTM (Takara, Japan) with the fluorescent quantitative PCR C1000 Touch Thermal Cycler (CFX96 Real-Time System, BioRad, United States). Each 10 μL reaction mixture consisted of nuclease-free water (2.5 μL), SYBR Premix ExTaq II (5.0 μL), primer (i.e. 0.25 μL each with 0.8 μM concentration), and diluted cDNA (2 μL). The PCR cycles were performed as: 94 °C for 3 min, followed by 94 °C for 30 s, 55 °C for 45 s and 72 °C for 1 min up to 40 cycles. All experiments were performed in triplicate. Relative fold expression changes and standard error were calculated using the 2-ΔΔCt method^44^. Each cDNA sample were normalized with endogenous reference gene i.e., Tubulin (from pigeon pea).

### Statistical analysis

R package and Microsoft excel was used to analyse the data.

## Supplementary Information

Supplementary Information 1.

Supplementary Information 2.

Supplementary Information 3.

Supplementary Information 4.

Supplementary Information 5.

Supplementary Information 6.

Supplementary Information 7.

Supplementary Information 8.

Supplementary Information 9.
