# Supplementary material for: Exploring the edible gum (galactomannan) biosynthesis and its regulation during pod developmental stages in clusterbean using comparative transcriptomic approach
Source: Sci Rep. 2021 Feb 17;11:4000. doi: 10.1038/s41598-021-83507-3 (PMC7890066; doi:10.1038/s41598-021-83507-3)
Supplement: Supplementary file 1 — Supplementary Information 1. [file 41598_2021_83507_MOESM1_ESM.pdf]

Supplementary table 1A. Quality assessment of the reads generated from RNA-Seq for the RGC-936 and M-83 Clusterbean genotypes at three pod development stages.

**Sample_name**	B2_R25_R1	C2_R25_R2	D2_M25_R1	E2_M25_R2	F2_R39_R1	G2_R39_R2	B4_M39_R1	C4_M39_R2	D4_R50_R1	E4_R50_R2	F4_M50_R1	G4_M50_R2
**accession**	SRR9176906	SRR9176907	SRR9176908	SRR9176909	SRR9176902	SRR9176903	SRR9176904	SRR9176905	SRR9176900	SRR9176901	SRR9176910	SRR9176911
**biosample_accession**	SAMN11927998	SAMN11927999	SAMN11928004	SAMN11928005	SAMN11928000	SAMN11928001	SAMN11928006	SAMN11928007	SAMN11928002	SAMN11928003	SAMN11928008	SAMN11928009
**Raw Reads Number**	29173972	23197384	29707907	25097319	24831694	19071241	20297178	24891294	20832904	21169939	25203583	23378824
**Raw Reads Length (bp)**	150	150	150	150	150	150	150	150	150	150	150	150
**GC (%)**	46	44	44	44	43	43	43	44	45	43	45	46
**Clean Reads Number**	29101816	23034482	29635244	25041511	24609162	19046620	20241886	24834382	20771122	21092650	24999623	23305877
**Clean Reads Length (bp)**	20-150	20-150	20-150	20-150	20-150	20-150	20-150	20-150	20-150	20-150	20-150	20-150
**GC (%)**	46	44	43	43	43	42	42	43	44	42	44	45
