# Supplementary material for: Exploring the edible gum (galactomannan) biosynthesis and its regulation during pod developmental stages in clusterbean using comparative transcriptomic approach
Source: Sci Rep. 2021 Feb 17;11:4000. doi: 10.1038/s41598-021-83507-3 (PMC7890066; doi:10.1038/s41598-021-83507-3)
Supplement: Supplementary file 5 — Supplementary Information 5. [file 41598_2021_83507_MOESM5_ESM.pdf]

Supplemental Table S3: Number of unigenes identified related to galactomannan biosynthesis at three developmental stages between two clusterbean genotypes (RGC-936 and M-83)

S.No.	Enzymes	R25-M25	R39-M39	R50-M50
1	Phosphomannomutase	4	11	9
2	Mannan endo-1,4-beta-mannosidase	21	24	23
3	Mannan synthase-like	6	2	2
4	UDP-glucose pyrophosphorylase	1	1	1
5	Alpha-galactosidase	44	54	53
6	UDP-Glycosyltransferase	-	36	39
7	Sucrose synthase	53	51	43
8	Cellulose synthase	165	161	156
9	Beta galactosidase	137	145	146
10	UDP-glucose 4 epimerase/UDP-galactose 4 epimerase	58	36	33
11	UDP-galactose transporter	1	8	8
12	Glucan endo-1,3-beta-D-glucosidase	19	8	98
13	Alpha-1,2-glucosyltransferase	-	3	3
14	Alpha-1,3-glucosyltransferase	4	9	9
15	Alpha-1,4 glucosidase	4	-	-
16	Beta glucosidae + Glucan endo-1,3-beta-D-glucosidase	43	211	206
17	Glucosyltransferase activity	135		127

Supplemental Table S5: List of primers used for qRT-PCR analysis

S. No.	Primer ID (Forward)	Primer sequence	Primer ID (Reverse)	Primer sequence
1	GluT_Ct_F	CCAGCATCAGCAGCCATATT	GluT_Ct_R	GCCCTTGCATCCATCTCTAAC
2	Man_Ct_F	GGGAGTGAAATGGGAGAAGAAG	Man_Ct_R	GGACTCATCCGCAGCAAATA
3	SucS_Ct_F	AACTAGGATCACTCTGGCATTG	SucS_Ct_R	TGGCCCTGCTGAGATTATTG
4	GlyT_Ct_F	CGTCCCTTCCTTTGTCTTCTAC	GlyT_Ct_R	CAATTGCCAACTCAGCACTATC
5	β-gal_F	TCGGATGCTGCCTGTTATATG	β-gal_R	TACCCTCTCTCTCTCTCTCTCT
6	UGE_F	GCCCTCTTCCATACCAAACTAC	UGE_R	GAATGCAATGTGAAACCAGACC
7	β-glu_F	CTCGTTGCAAGCACCTTCTA	β-glu_R	GGTTCTGCTAATGGACAACTCT
8	α-gal_F	GGGCTAGAAGTGTGGGAAATAG	α-gal_R	TCCAGCATAAGAAGCCCATTTA
9	Csyn_Ct_F	GCAGACAGCAGGAAGAATACA	Csyn_Ct_R	GTATGGCTACAAGGAAGGGAAG
10	bhlh_Ct_F	ATGGGAGCAGAGTTGAGATTAG	bhlh_Ct_R	CAATGAAAGAGTCGAGCCAAAG
11	NAC_Ct_F	CAATAGTGTGGGCTCCAAGAA	NAC_Ct_R	CTTAGTATCCAATCCCGCTGAC
12	G2like_Ct_F	GTGAAGGAGGTTCCTTATCCTG	G2like_Ct_R	CTTGGCATATGTGGGCATTTC
13	bzip_Ct_F	GGTCATCTCAGCATAAGAAGCTA	bzip_Ct_R	TGGTACATCAAGGGCGTAATC
14	LBD_Ct_F	GGGTGAGTAACGCGTAAGAA	LBD_Ct_R	GCCTCACCAACTAGCTAATCA
15	Myb_Ct_F	GCTTGGGAAGGAGTAGGTAATAAA	Myb_Ct_R	GTCATGCACGAGAAGTTGGA
