# Supplementary figures and images for: Exploring the edible gum (galactomannan) biosynthesis and its regulation during pod developmental stages in clusterbean using comparative transcriptomic approach
Source: Sci Rep. 2021 Feb 17;11:4000. doi: 10.1038/s41598-021-83507-3 (PMC7890066; doi:10.1038/s41598-021-83507-3)

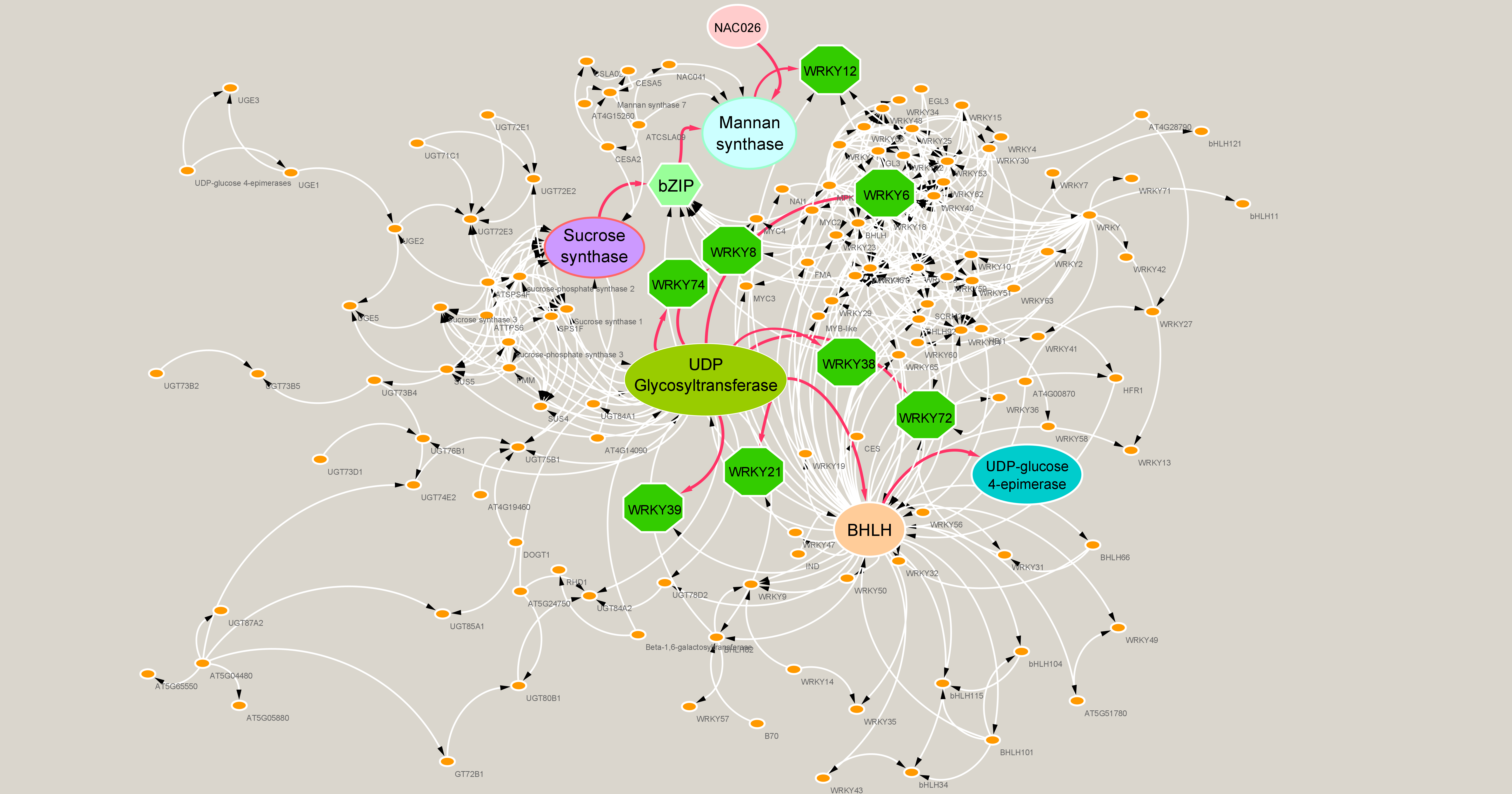

Supplement: Supplementary file 8 — Supplementary Information 8. [file 41598_2021_83507_MOESM8_ESM.tiff]
