# Supplementary material for: Exploring the edible gum (galactomannan) biosynthesis and its regulation during pod developmental stages in clusterbean using comparative transcriptomic approach
Source: Sci Rep. 2021 Feb 17;11:4000. doi: 10.1038/s41598-021-83507-3 (PMC7890066; doi:10.1038/s41598-021-83507-3)
Supplement: Supplementary file 9 — Supplementary Information 9. [file 41598_2021_83507_MOESM9_ESM.docx]

Title:

**List of Supplementary Table 1-6 for the paper entitled “**Exploring the edible gum (galactomannan) biosynthesis and its regulation during pod developmental stages in cluster bean using comparative transcriptomic approach**”**

Sandhya Sharma^a^, Anshika Tyagi^a^, Harsha Srivastava^a^, Ramakrishna G^a^, Priya Sharma^a^, Amitha Mithra Sevanthi^a^, Amolkumar U Solanke^a^, Ramavtar Sharma^b^, Nagendra Kumar Singh^a^, Tilak Raj Sharma^a,c^,Kishor Gaikwad^a^*

1. ICAR-National Institute for Plant Biotechnology, New Delhi, India
2. ICAR-Central Arid Zone Research Institute, Jodhpur, India
3. DBT-National Agri-Food Biotechnology Institute, Mohali, India

***Corresponding Author** Tel. 01125841787; ext. 246. Email: [kish2012@gmail.com](mailto:kish2012@gmail.com)

**Supplementary Table 1A.** Quality assessment of the reads generated from RNA-Seq for the RGC-936 and M-83 Clusterbean genotypes at three pod development stages.

**Supplementary Table 1B.** Differentially expressed genes (DEGs at p­< 0.01) for all 15 combinations (R25-M25, R39-M39, R50-M50, R25-M39, R25-M50, R39-M50, M25-R39, M25- R50, M39-R50, R25-R39, R25-R50, R39-R50, M25-M39, M25-M50, M39-M50) between two clusterbean genotypes RGC-936 and M-83 at three pod development stages (25DAF, 39DAF and 50DAF).

**Supplementary Table 2A and 2B.** NR annotation **(**at p< 0.01) for all 15 combinations (R25-M25, R39-M39, R50-M50, R25-M39, R25-M50, R39-M50, M25-R39, M25-R50, M39- R50, R25-R39, R25-R50, R39-R50, M25-M39, M25-M50, M39-M50) between two clusterbean genotypes RGC-936 and M-83 at three pod development stages (25DAF, 39DAF and 50DAF).

**Supplementary Table S3**. Unigenes identified related to galactomannan biosynthesis at three developmental stages between two genotypes

**Supplementary Table S4.** KEGG and COG annotation **(**at p< 0.01) for all 15 combinations (R25-M25, R39-M39, R50-M50, R25-M39, R25-M50, R39-M50, M25-R39, M25-R50, M39- R50, R25-R39, R25-R50, R39-R50, M25-M39, M25-M50, M39-M50) between two clusterbean

genotypes RGC-936 and M-83 at three pod development stages (25DAF, 39DAF and 50DAF).

**Supplementary Table S5.** List of primers used for qRT-PCR analysis

**Supplementary Table S6.** Transcription factor annotation **(**at p< 0.01) for all 15 combinations (R25-M25, R39-M39, R50-M50, R25-M39, R25-M50, R39-M50, M25-R39, M25- R50, M39-R50, R25-R39, R25-R50, R39-R50, M25-M39, M25-M50, M39-M50) between two clusterbean genotypes RGC-936 and M-83 at three pod development stages (25DAF, 39DAF and 50DAF).
